# Biological Clock Depends on Many Parts Working Together

**DOI:** 10.1371/journal.pbio.0000023

**Published:** 2003-09-15

How do people subjected to the endless dark days of winter in the far northern latitudes maintain normal daily rhythms? Though many might feel like hibernating, a highly regulated internal system keeps such impractical yearnings in check. From fruit flies to humans, nearly every living organism depends on an internal clock to regulate basic biological cycles such as sleep patterns, metabolism, and body temperature. And that clock runs on similar molecular mechanisms.

Specific clusters of neurons in the brain are known to control the biological clock. Scientists believed these brain “clock cells” function as independent units. But new research described in this issue shows that the neurons do not act in isolation; rather, they collaborate with other neurons in a cell-communication network to sustain the repeating circadian rhythm cycles.

Clock cells within the brain maintain an organism's circadian rhythms, even in the absence of cyclical environmental signals like light, in a state scientists call “free running.” Though it has long been clear that the circadian rhythms of an organism persist under such free-running conditions (for example, constant darkness), it was thought that the gene-expression patterns within the cells governing these biorhythms did not require any external, or extracellular, signals to continue ticking. In experiments described here, Michael Rosbash and his colleagues show that the key brain clock cells in fruit flies (*Drosophila*), called *ventral lateral neurons*, do indeed support the fly's circadian rhythms during periods of constant darkness and that the molecular expression patterns associated with these rhythms continue to cycle as well within other clock cells. These sustained expression patterns, however, require intercellular communication between different groups of brain clock cells.

In other words, the ventral lateral neurons do not act alone. When the molecular clock machinery was manipulated so that only the ventral lateral neurons were active, the fly's circadian rhythms were not sustained, suggesting the rhythms depend on other neuronal groups as well. The researchers also demonstrate that the persistence of normal cycling during constant darkness depends on a protein (called PDF) secreted by the ventral lateral cells.

The PDF neuropeptide protein was thought to connect the molecular expression pattern of the ventral lateral neurons with the manifestation of circadian rhythms, but the researchers found evidence of a larger influence. When mutant flies lacking a functional PDF gene were exposed to constant darkness, the molecular expression patterns gradually stopped. The scientists say this suggests that the ventral lateral neurons and the PDF protein it produces help coordinate the entire neural network that underlies circadian rhythms.

**Figure:**
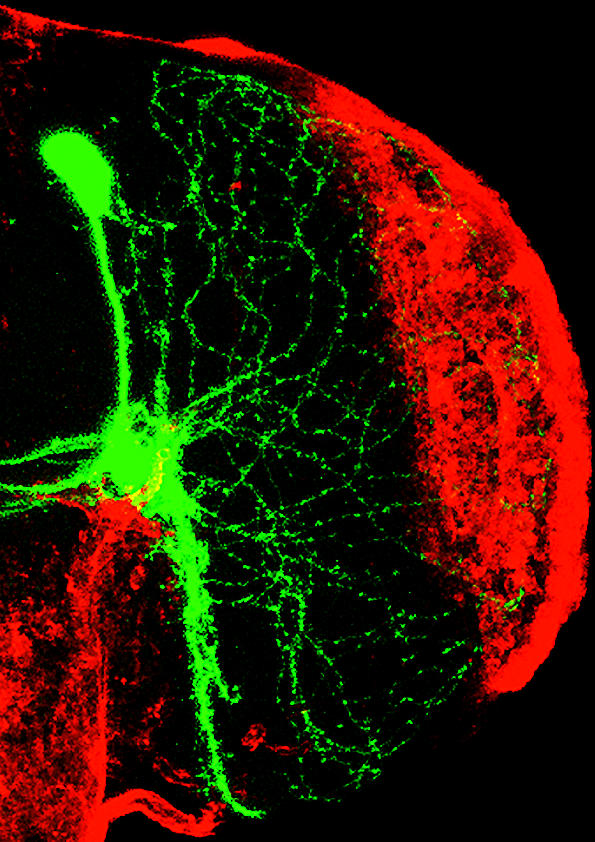
Drosophila lateral neuron (green)

